# M1 Macrophage exosomes MiR‐21a‐5p aggravates inflammatory bowel disease through decreasing E‐cadherin and subsequent ILC2 activation

**DOI:** 10.1111/jcmm.16348

**Published:** 2021-02-10

**Authors:** Jiaxi Lu, Deliang Liu, Yuyong Tan, Feihong Deng, Rong Li

**Affiliations:** ^1^ Department of Gastroenterology Research Center of Digestive Disease The Second XiangYa Hospital Central South University Changsha China

**Keywords:** E‐cadherin, group 2 innate lymphoid cell, macrophage‐derived exosome, MiR‐21a‐5p, ulcerative colitis

## Abstract

Abnormal immune regulation is a key feature of the complex pathogenic mechanism of ulcerative colitis (UC). In particular, macrophages and group 2 innate lymphoid cells (ILC2s) are important components of natural immunity that have been shown to play important roles in the pathogenesis of UC, as well as decreased E‐cadherin expression on the colonic mucosa. However, it remains unclear how these components interact with each other. In this study, we investigated the molecular mechanisms of UC mediated by macrophage‐derived exosomes. We showed for the first time that miR‐21a‐5p expression is increased in the peritoneal exosomes of mice with dextran sulphate sodium induced enteritis and that miR‐21a‐5p expression correlates negatively with E‐cadherin expression in enterocytes. Moreover, we confirmed that miR‐21a‐5p was mainly derived from M1 macrophages and demonstrated that KLRG1, a surface inhibitory receptor on ILC2s, participated in excessive ILC2 activation in UC by promoting GATA‐3. In conclusion, our results suggest molecular targets and provide a theoretical basis for elucidating the pathogenesis of UC and improving its treatment.

## 1 INTRODUCTION

Ulcerative colitis (UC) is a type of inflammatory bowel disease that mainly affects the rectum and colon and is characterized by the destruction of colonic mucosal barrier function (barrier disruption) as well as repeated diarrhoea containing mucus, pus and blood. The incidence of UC is rising worldwide, and the disease has become a significant nationwide health problem in China.^1^, ^2^ UC has a complex and multifactorial pathogenesis that includes genetic predisposition, epithelial barrier dysfunction, dysregulated immune responses, pathogenic infection and environmental factors. In addition, the intestinal mucosal immune system plays an important role in the occurrence, progression and outcome of UC ^3^ and abnormal immune regulation is a key feature of its pathogenesis.

Macrophages are an important component of natural immunity that form part of the first line defence in intestinal mucosa and have been shown to play an important role in the initiation of UC.^3^, ^4^, ^5^, ^6^ In humans, macrophages are classified into two subsets with seemingly different functions: M1 macrophages, also known as ‘classically activated’ macrophages, are mainly involved in biological processes that promote inflammation and inhibit tumours, whereas M2 macrophages, also known as ‘selectively activated’ macrophages, play important roles in tumour progression, parasite control and tissue repair.^7^ The pathogenic M1 subset is significantly more prevalent in colons with inflammation induced by dextran sodium sulphate (DSS), and this difference is consistently observed in most locations.^9^, ^10^ However, studies have shown that the phenotype of macrophages can be switched depending on the presence of different cytokines in their microenvironment.^8^

Pharmacotherapy has been shown to ameliorate experimental inflammatory bowel disease by inducing a switch from M1 to M2 macrophages,^11^ whereas extracellular vesicles derived from bone marrow mesenchymal stem cells have been found to attenuate DSS‐induced UC by promoting M2 macrophage polarization.^12^ Previously, we developed a new immunotherapeutic strategy for treating UC that uses dual‐targeting nanoparticles to deliver miR‐146b to intestinal macrophages, where they induce M2 polarization, activate the IL‐10‐dependent STAT3 pathway and thereby promote the mucosal repair of DSS‐induced enteritis.^13^ However, it remains unclear whether abnormally polarized peritoneal M1 macrophages are involved in intestinal mucosal damage.

Exosomes are a heterogeneous group of cell‐derived membranous structures that originate from the endosomal system or are shed from the plasma membrane. Importantly, exosomes are present in biological fluids and are involved in multiple physiological or pathological processes.^14^ For instance, serum exosomes isolated from DSS‐induced mice have been shown to induce p38 and ERK phosphorylation as well as the production of tumour necrosis factor α (TNF‐*α*), suggesting that exosomes may play an important role in the pathogenesis of UC.^15^ Moreover, the oral administration of autologous exosomes from the healing phase could represent a safe and effective personalized approach for treating UC.^16^ However, it is not yet clear whether dysregulated M1 macrophage‐mediated immune responses play a pathogenic role in UC via exosomes.

The innate immune system provides the first line of defence against invading pathogens as well as important cues for the development of adaptive immunity. Innate lymphoid cells (ILCs) are a new class of lymphocytes that have recently been discovered to play an important role in innate immunity at the mucosal barrier and promote the establishment and maintenance of adaptive immunity.^17^ According to their phenotypes and secreted cytokines, ILCs can be divided into three categories: ILC1, ILC2 and ILC3. ILC2s are important source of Th2‐type cytokines and play important roles in the activation and maintenance of Th2‐type immunity.^18^ DSS‐induced colitis displays a skewed Th2 immunity characterized by low levels of IFN‐*γ* and TNF‐*α* but high levels of IL‐4, IL‐6, IL‐10, IL‐17A and IL‐17F.^19^, ^20^ In addition, the level of Th2 cytokines has been associated with the severity of UC and poor prognosis; therefore, IL‐13 has been suggested as a new target for host‐directed therapies against UC.^21^, ^22^, ^23^ Forkel et al also found that UC inflamed colons contain significantly more ILC2s,^24^ indicating that these cells may participate in the pathogenesis of UC by promoting Th2 immunity.

We used miRWalk to search potential target gene of miR‐21a‐5p. The results showed that Cdh1, which is the gene that express E‐cadherin, was one of the possible target gene (*P* =0.846) and they may interact through 3'UTR. Combined with the result of mouse intraperitoneal exsosomes high throughout sequencing, which indicated that miR‐21a‐5p had one of the most significant expression change between DSS‐induced enteritis mice and healthy mice, we speculate that miR‐21a‐5p may attenuate ulcerative colitis by decrease the expression of E‐cadherin. Besides, the correlation between E‐cadherin and KLRG1 and the correlation between KLRG1 and GATA‐3 have been proved by several studies. KLRG1 is a well‐known marker of NK cells that is also expressed on ILC2s, and KLGR1 binds with its ligand, E‐cadherin, indicates an immunosenescent phenotype. Therefore, in this study, we aimed to clarify the role and mechanism of macrophages and ILC2s in the pathogenesis of UC to provide potential targets and a theoretical basis for the development of new drugs to prevent and treat UC.

## 2 MATERIALS AND METHODS

### 2.1 Human samples

Peripheral blood mononuclear cells (PBMCs), serum and colon samples were obtained from patients with active UC (n = 30) and healthy volunteers (n = 30) with approval from the Institute Research Medical Ethics Committee of Xiangya Second Hospital (Hunan, China). Colon samples were collected during colonoscopy biopsies performed in the Department of Gastroenterology. Written informed consent was obtained from all participants.

### 2.2 Mice and DSS‐induced colitis

Male C57BL/6 mice (7 to 8 weeks old, 20 ± 0.5 g) were purchased from the Medical Experimental Animal Center of Xiangya Second Hospital for use in our experiments with approval from the Animal Experimentation Committee of Xiangya School of Medicine, Central South University. Thirty male mice were maintained on a 12‐hour/12‐hour light/dark cycle in a temperature‐controlled room (24°C). Firstly, mice were fed with normal food and water for one week to adapt. Then, they were randomly divided into two equal groups: a) DSS‐induced colitis group and a control group. The DSS‐induced colitis group received 3% DSS (MP Biomedicals, Ohio, USA) dissolved in drinking water for 6 days, followed by normal drinking water for 5 consecutive days. Control group was the same as the experimental group but with normal drinking water for 11 consecutive days. Weight change was recorded every day. Model success and colitis severity were evaluated using the weight loss and disease activity index (DAI).

### 2.3 Exosome extraction

After the mice had been anaesthetized and killed, 5 mL of RPMI‐1640 was intraperitoneally injected and the abdomen was gently kneaded for 2‐3 minute. The plunger of the syringe was gently withdrawn and pushed several times and then withdraw to obtain peritoneal wash fluid, which was subjected to gradient centrifugation as follows: 1000 *g* for 10 minutes; supernatant removed; 2000 *g* for 10 mins; supernatant removed; 10 000 *g* for 30 minutes; supernatant removed; supernatant filtered with a 0.22 μm filter; 100 000 *g* for 120 minutes; and purified exosomes harvested from the precipitate. Exosomes were identified by particle size analysis using transmission electron microscopy and morphology (Figure S1).

### 2.4 Cells

FHC and THP‐1 cell lines were purchased from the American Type Culture Collection (ATCC; Manassas, VA, USA). Complete medium was prepared from 90% RPMI‐1640, 10% foetal bovine serum (FBS) and 1% penicillin/streptomycin (100 U/mL) at 37°C with 5% CO_2_. To produce the DSS‐induced inflammation model in vitro, FHC cells were starved for 12 hours, treated with 1% DSS for 4 hours and then incubated with reduced serum culture medium at 37°C for 4 hours. Clophosome (FormuMax Scientific, California, USA) was used to achieve macrophage depletion.

### 2.5 Microphage differentiation

THP‐1 cells were differentiated into M0, M1 and M2 macrophages as described previously (25). Briefly, THP‐1 cells were treated with 200 nM phorbol 12‐myristate 13‐acetate (PMA) for 24h until they became adherent, indicating that an M0 phenotype had been induced (Figure S2). To produce M2 cells, the M0 macrophages were treated with 20 ng/mL of IL‐4 and IL‐13 for a further 18 hours whereas M1 macrophages were induced by incubation with 100 ng/mL of LPS and 20 ng/mL of IFN‐γ. The control group was treated with same amount of solvent, which is phosphate buffer saline, at the same time‐point.

### 2.6 Co‐culture of exosomes and FHC cells

Exosomes were isolated as described above. The exosomes were added to FHC cells for 12 hours of incubation at 37℃. The PKH 26‐labelled exosomes displayed red colour. Cytoskeleton was stained with phalloidin (green), and the cell nuclei were stained with DAPI (blue). Microscopic observation was performed at 0 hours, 4 hours and 12 hours.

### 2.7 Reverse transcription‐polymerase chain reaction (RT‐PCR)

RNA was extracted using TRIzol (Thermo Fisher Scientific, New York, USA) according to the manufacturer's protocol. Real‐time PCR was conducted using an iScriptTM two‐step RT‐PCR kit with SYBR Green (Invitrogen) and gene‐specific primers (Table S1). PCR was performed in triplicate on Roche LightCycler® 480 (Roche, Basel, Swiss) with the following cycling parameters: 95 ℃ (1 minute) followed by 40 cycles of 95°C (15 seconds) and 60°C (60 seconds). The qRT‐PCR data were quantified using the 2‐^△△^Ct method.

### 2.8 Flow cytometry

M0, M1 and M2 cells were profiled by flow cytometry using a FACS Canto II system (BD, Franklin Lakes, NJ, USA). M1 and M2 cells were verificated using CD 86 and CD206 as markers, respectively. CD68 and CD11b were used as M0 markers. (Figure S3) ILC2s were sorted using Lin (CD3, CD4, CD19, CD11c, CD56, FceR1)‐, CD127 + and CRTH2 + as markers (Figure S4).

### 2.9 ELISA

Plasma IL‐4, IL‐5, IL‐6, IL‐11, IL‐13, IL‐22, TNF‐α and IFN‐γ levels were measured using an ELISA kit (Shanghai ExCell Biology, China) according to the manufacturer's protocols. Concentrations were calculated using a standard curve.

### 2.10 Statistical analysis

SPSS for Windows (Version 21.0, SPSS Inc, Illinois, USA) was used for statistical analysis. Statistical differences between the experimental group and control group were assessed with the one‐way ANOVA test. Correlations were evaluated using the Spearman rank correlation coefficient. Statistical significance was set at *P* <0.05.

## 3 RESULTS

### 3.1 Macrophages produce exosome‐derived miR‐21a‐5p

**FIGURE 1:**
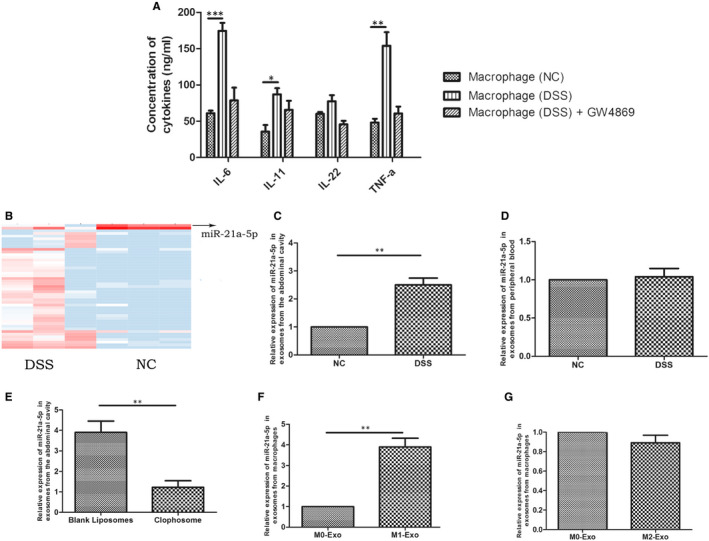
miR‐21a‐5p increased in intraperitoneal M1 macrophage of DSS‐induced enteritis mice. (A) The concentration of IL‐6, IL‐11, IL‐22 and TNF‐*α* in DSS‐induced enteritis mice intraperipheral macrophage group (n = 15) was significantly higher than control group (n = 15), but showed no difference in GW4869 group; (B‐C) The expression of miR‐21a‐5p significantly increased in intraperitoneal macrophage of DSS‐induced enteritis mice (n = 15), (D) but showed no difference in peripheral blood serum of mice (n = 15). (E) Intraperitoneal macrophage miR‐21a‐5p significantly decreased in Clophosome group (n = 15). (F) The expression of miR‐21a‐5p significantly higher in M1 group than M0 group, (G) but showed no difference between M2 group and M0 group. ^**^ *P* < 0.01

First, in order to confirm whether intraperitoneal macrophages attenuate ulcerative colonitis through exosomes, we co‐cultured mouse colonic epithelial cells with intraperitoneal macrophages from healthy mice, mice with DSS‐induced enteritis or mice with DSS‐induced enteritis treated with the exosome inhibitor, GW4869. Co‐culture with intraperitoneal macrophages from mice with DSS‐induced enteritis significantly increased the levels of inflammatory factors (IL‐6, IL‐11, IL‐22, TNF‐α) in colonic epithelial cells compared to macrophages from healthy mice, whereas GW4868 eliminated this difference (Figure 1A). To identify potential factors involved in this mechanism, we performed high‐throughput sequencing on intraperitoneal exosomes extracted from mice with DSS‐induced enteritis and healthy mice. Significantly higher miR‐21a‐5p levels were observed in the intraperitoneal exosomes extracted from mice with DSS‐induced enteritis mice than in the control group, whereas miR‐21a‐5p levels in peripheral blood remained similar (Figure 1B‐D).

Next, we determined whether miR‐21a‐5p was secreted by macrophages by examining the numbers of intraperitoneal macrophages and miR‐21a‐5p expression in intraperitoneal exosomes from mice with DSS‐induced enteritis pre‐treated with Clophosome, which can efficiently exhaust macrophages. Clophosome not only depleted the levels of intraperitoneal macrophages, but also significantly decreased miR‐21a‐5p expression in intraperitoneal exosomes (Figure 1E). Therefore, we compared miR‐21a‐5p expression in M1‐ and M2‐derived exosomes by inducing THP‐1 cells to become M0 and then M1 or M2 macrophages. The efficacy of M0 induction was confirmed by flow cytometry, which indicated that 90% of cells were successfully induced. Moreover, comparing the expression of miR‐21a‐5p in the supernatant of M1 and M2 exosomes revealed that M1 exosomes displayed significantly higher miR‐21a‐5p expression than M0 exosomes (Figure 1F), whereas M2 exosomes showed no significant difference (Figure 1G).

### 3.2 Exosome‐derived miR‐21a‐5p aggravates DSS‐induced enteritis by decreasing E‐cadherin expression

**FIGURE 2:**
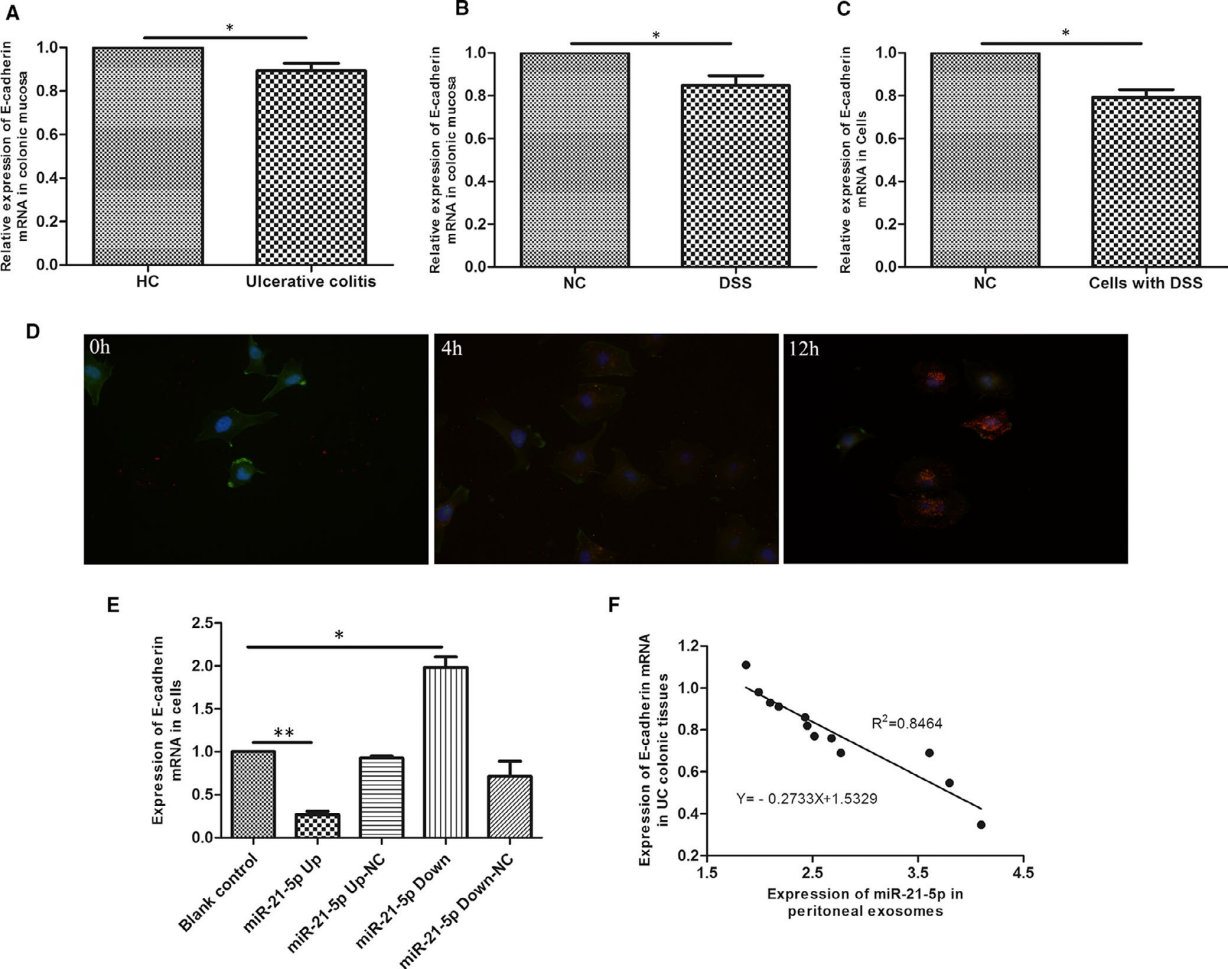
E‐cadherin was down‐regulated in UC or DSS‐induced enteritis. E‐cadherin mRNA decreased in (A) intestinal mucosa of UC patients (n = 30), (B) intestinal mucosa of DSS‐induced enteritis mice (n = 15) and (C) DSS‐treated colonic epithelial cells. (D) Microscopic analysis of the internalization of the pre‐labelled exosomes in different time (0h, 4h, 12h). The exosomes displayed red colour. Cytoskeleton was stained with in green, and the cell nuclei were stained in blue, 400×. (E)There was a negative correlation between miR‐21a‐5p and E‐cadherin in colonic epithelial cells with different expression of miR‐21a‐5p. (F) Correlation analysis between intraperitoneal macrophage miR‐21a‐5p and E‐cadherin in intestinal mucosa from DSS‐induced enteritis mice (n = 15). The slope is −0.2733. *R^2^* = 0.8464. ^*^ *P* < 0.05, ^**^ *P* < 0.01. HC: healthy volunteers control group

After we confirmed that miR‐21a‐5p in M1 exosomes could attenuate DSS‐induced enteritis, further investigation of the target gene of miR‐21a‐5p was made. Using miRwalk, a well‐established target gene prediction website, to search potential target gene of miR‐21a‐5p. The results showed that Cdh1, which is the gene that express E‐cadherin, was one of the possible target gene. Subsequently, we compared the E‐cadherin expression in colonic epithelial cells from patients with UC, colonic epithelial cells from mice with DSS‐induced enteritis, and DSS‐treated FHC cells using RT‐PCR. Interestingly, E‐cadherin mRNA expression was lower in all three experimental groups than in their respective control groups (Figure 2A,C). Co‐culture experiment showed that red signals located apart from the FHC cells (Cytoskeleton was stained with phalloidin, green signals; nuclei stained with DAPI, blue signals) at 0 hours and then start to merge at 4 hours. Finally at 12 hours, almost all red signals located inside of the FHC cells, indicating that FHC cells internalize the exosomes contained miR‐21a‐5p secreted by M1 cells. Therefore, we investigated whether a correlation existed between miR‐21a‐5p and E‐cadherin by producing stable miR‐21a‐5p‐overexpressing and ‐silenced FHC cell strains and examining E‐cadherin mRNA expression (Figure S5A). We found that miR‐21a‐5p up‐regulation decreased E‐cadherin mRNA expression, whereas its down‐regulation increased E‐cadherin mRNA expression (Figure 2E). In addition, correlation analysis revealed a negative correlation between the intraperitoneal macrophage‐derived exosomes of mice with DSS‐induced enteritis and E‐cadherin expression (R^2^ = 0.8464; Figure 2F).

### 3.3 Absence of E‐cadherin promotes ILC2 activation via KLRG1/GATA‐3

**FIGURE 3:**
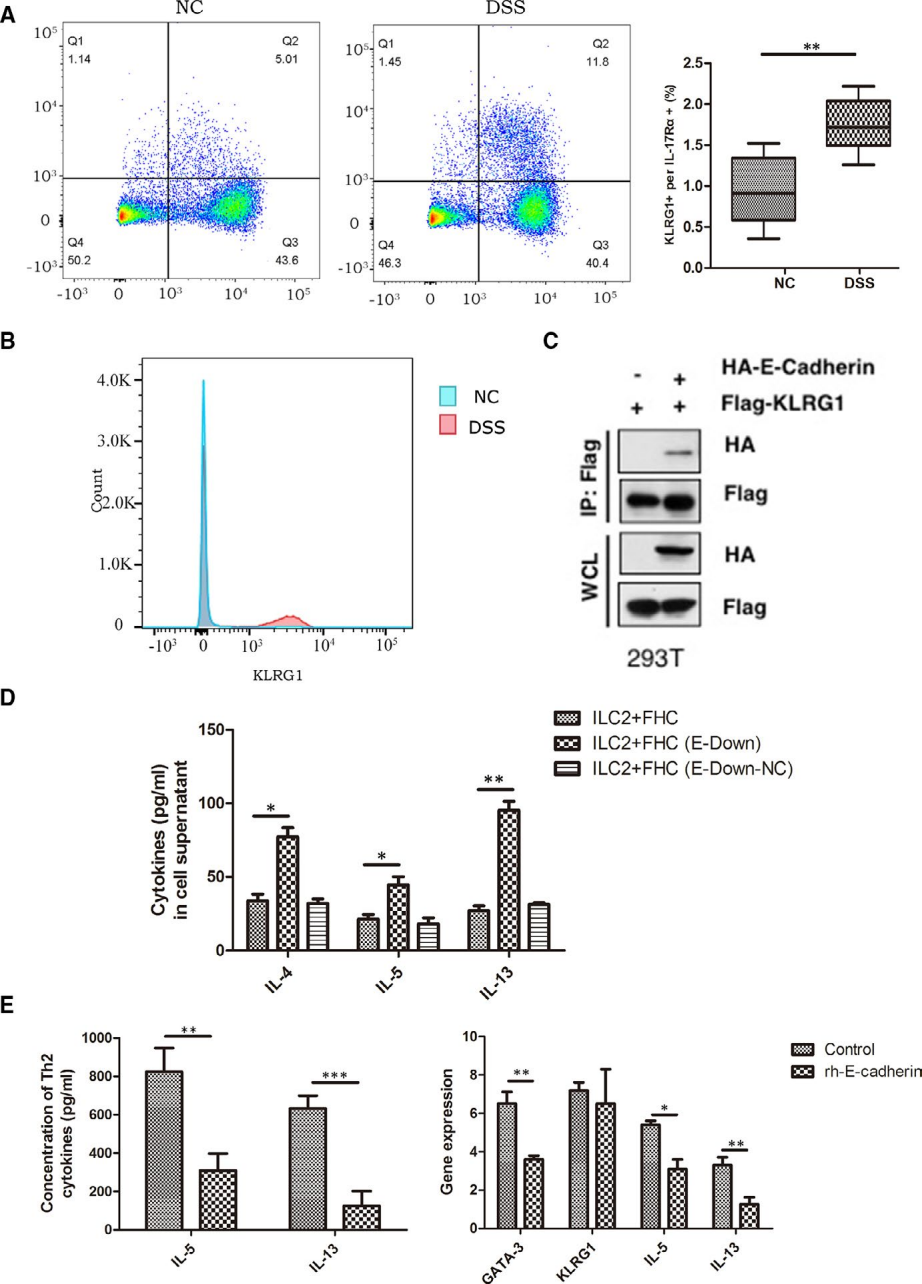
E‐cadherin may inhibit the activation of ILC2 through KLGR1 and GATA‐3 in spleen macrophage cell of DSS‐induced enteritis mice. (A)KLRG1^+^ cells proportion and (B)KLRG1^+^ cells expression absolute value were significantly higher than control group; (C)Co‐IP showed interaction between E‐cadherin and KLRG1; (D) Colonic epithelial cells with down‐regulated E‐cadherin had a significant increase of Th2 type cytokines concentration after co‐cultured with ILC2. (E)After treated with rh E‐cadherin, in vitro activated ILC2 increased significantly less IL‐5 and IL‐13, and the expression of GATA‐3, IL‐5 and IL‐13 mRNA also decreased in ILC2. ^*^ *P* < 0.05, ^**^ *P* < 0.01, ^***^ *P* < 0.001

Because of the relationship between E‐cadherin and ILC2, we consequently speculated if low expression of E‐cadherin may effect ILC2. Next, we performed flow cytometry on spleen lymphocytes from mice with DSS‐induced enteritis to identify changes in their expression of KLRG1. Interestingly, mice with DSS‐induced enteritis displayed a significantly higher number and proportion of KLRG1‐positive cells than the control group (Figure 3A,B). Therefore, we performed immunoprecipitation assays on HEK293T cells transfected with Flag‐KLGR1 and HA‐E‐cadherin 36 hours earlier and positively identified an interaction between E‐cadherin and KLRG1 (Figure 3C).

Finally, we validated the inhibitory effect of E‐cadherin on ILC2s by co‐culturing E‐cadherin‐silenced colonic epithelial cells (Figure S5B) with newly sorted ILC2s and examining Th2 cytokine levels. Silencing E‐cadherin was found to increase Th2 cytokine levels (Figure 3D), whereas newly sorted ILC2s treated with E‐cadherin to induce in vitro activation secreted significantly less IL‐5 and IL‐13 and expressed lower levels of GATA‐3, IL‐5 and IL‐13 mRNA (Figure 3E). Together, these results suggest that E‐cadherin may inhibit the activation of ILC2s via KLRG1/GATA‐3.

### 3.4 ILC2s and Th2 immunity are activated in UC

**FIGURE 4:**
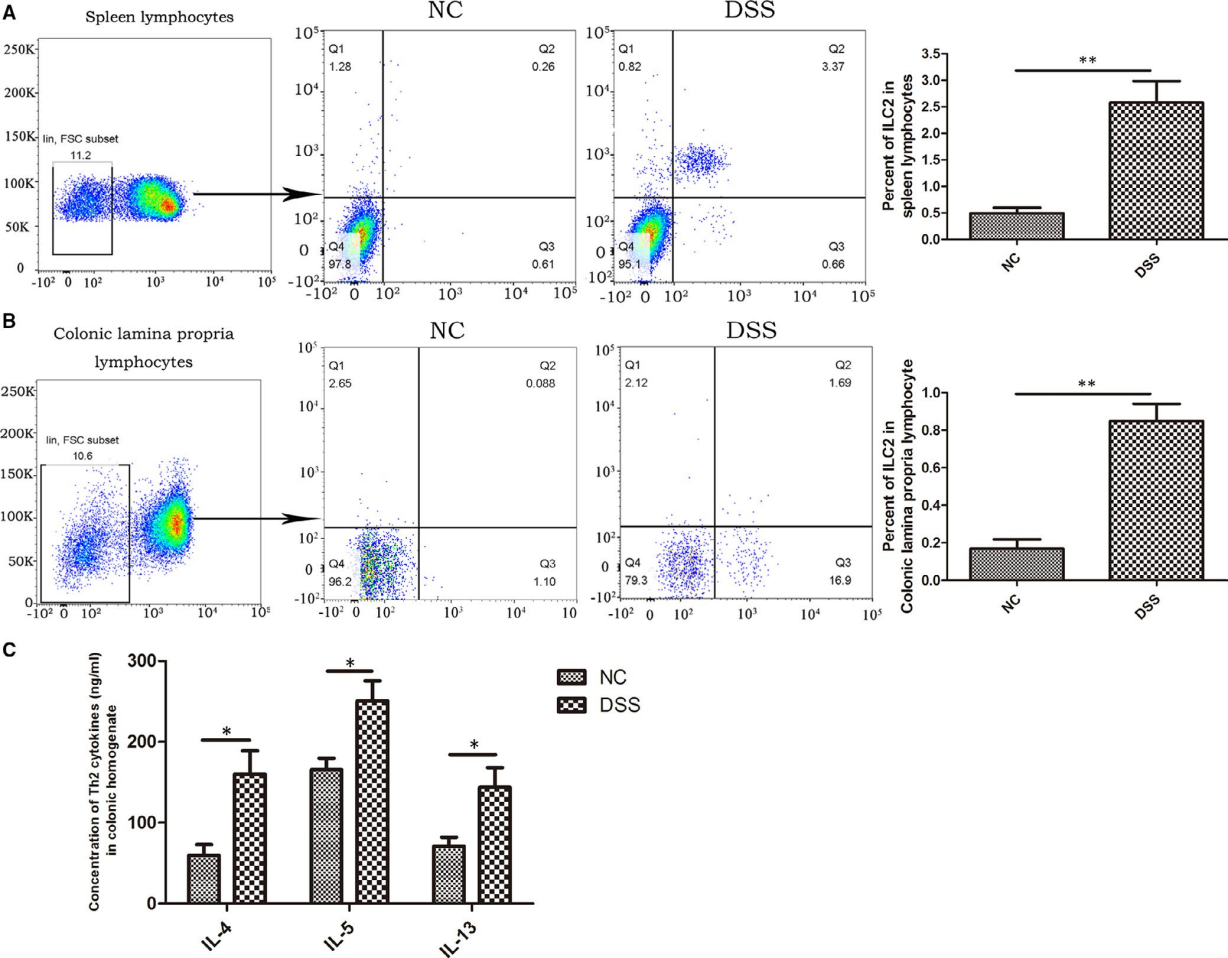
ILC2 proportion increased in UC. ILC2 proportion increased in (A) spleen macrophages, (B) colonic mucosa and lamina macrophages in DSS‐induced enteritis mice (n = 15) compared to control group (n = 15); (C) Th2 cytokines were significantly higher in colon tissue than in the control group. ^*^ *P* < 0.05, ^**^ *P* < 0.01

To elucidate the role that ILC2s play in UC, we evaluated the change in proportion of ILC2s in the spleen lymphocytes of mice with DSS‐induced enteritis using flow cytometry. A significant increase was observed in the proportion of ILC2s in the spleen and intestinal mucosa lamina propria lymphocytes of mice with DSS‐induced enteritis compared to the control group (Figure 4A‐B). In addition, we investigated the change in the proportion of Th2 cytokines in the intestinal mucosa of mice with DSS‐induced enteritis using ELISA, finding that the mice with DSS‐induced enteritis displayed significantly higher IL‐4, IL‐5 and IL‐13 levels than the control group (Figure 4C).

Therefore, we sought to evaluate the change in the proportion of ILC2s in the peripheral blood of healthy volunteers and patients with active UC as well as the expression of Th2 cytokines (IL‐4, IL‐5 and IL‐13) in peripheral blood serum and the mRNA expression of Th2 cytokines in the intestinal mucosa. No significant difference was observed between the proportion of ILC2s or Th2 cytokine expression in the two groups (Figure S6A); however, patients with active UC showed significantly higher Th2 cytokine levels than healthy volunteers (Figure S6B).

## 4 DISCUSSION

**FIGURE 5:**
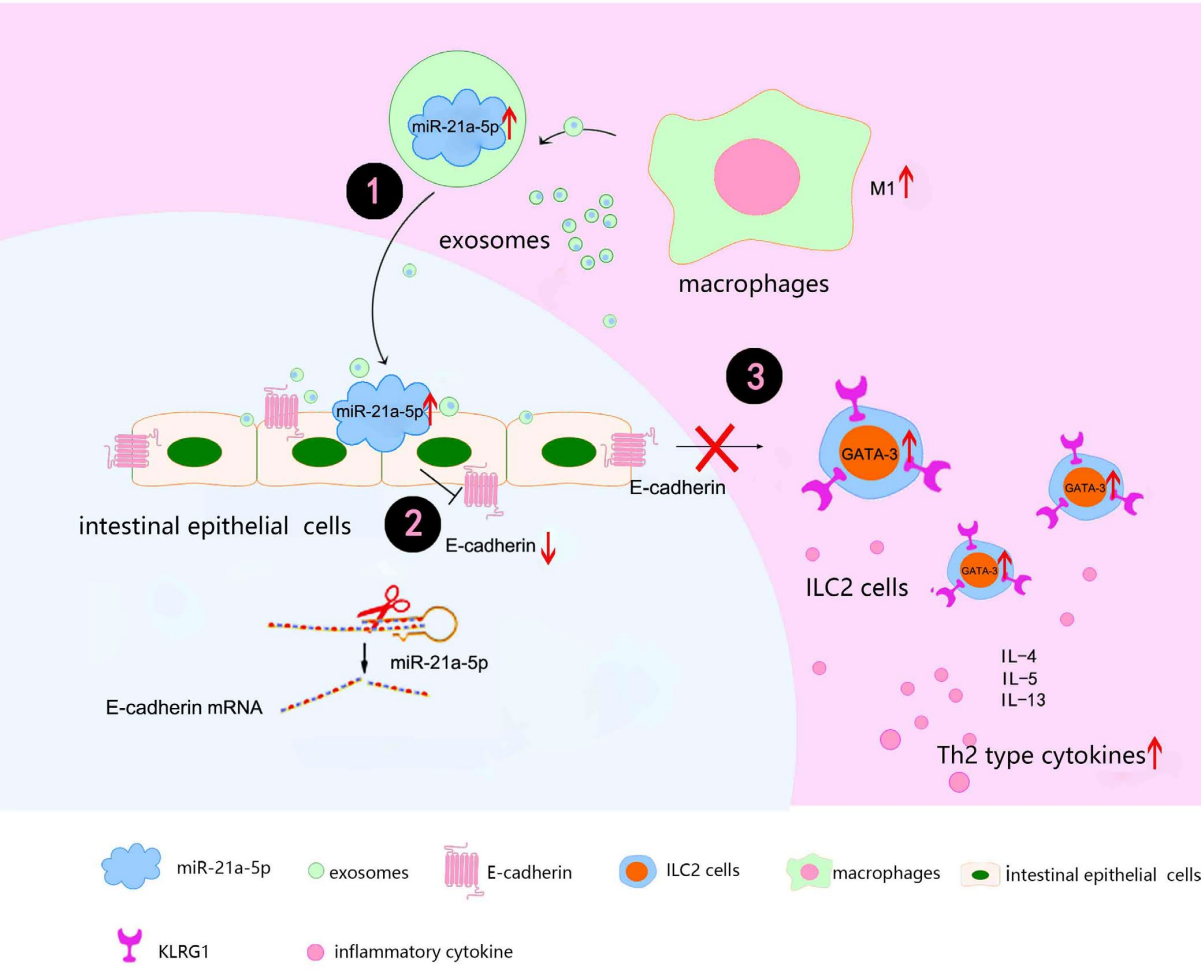
Model of how M1 macrophage exosomes miR‐21a‐5p aggravates inflammatory bowel disease through decreasing E‐cadherin and subsequent ILC2 activation

Cell adhesion molecules mediate adhesion and binding between cells or between cells and the extracellular matrix. E‐cadherin is a type of cell adhesion molecule that plays an important role in maintaining integrity by mediating mutual aggregation between cells ^25^, ^26^; however, its dysregulation has been shown to participate in the pathogenesis and remission of UC.^25^, ^26^, ^27^ In particular, the down‐regulation of E‐cadherin expression leads to the destruction of the intestinal mucosal barrier,^27^ which manifests clinically as repeated bloody diarrhoea. The findings of this study confirmed that E‐cadherin expression was significantly down‐regulated in intestinal mucosa with UC; however, it remains unclear whether abnormally polarized macrophages can regulate E‐cadherin expression in the intestinal mucosa or how this process is initiated. We demonstrated that exosome‐derived miR‐21a‐5p can be taken up by intestinal epithelial cells and regulate E‐cadherin expression and that miR‐21a‐5p was mainly derived from exosomes secreted by M1 macrophages. Therefore, miR‐21a‐5p may target E‐cadherin and result in the destruction of the intestinal mucosal epithelium, thus suggesting a novel mechanism via which abnormally polarized macrophages lead to the destruction of the intestinal mucosal barrier via E‐cadherin (Figure 5).

KLRG1 is a well‐known marker of NK cells that is also expressed on ILC2s and whose binding with its ligand, E‐cadherin, indicates an immunosenescent phenotype. As a key feature of mature ILC2s, KLRG1 plays a critical role in maintaining ILC2 cell function, whereas reduced KLRG1 expression or binding with E‐cadherin has been shown to induce excessive ILC2 activation and Th2‐type immunity.^28^, ^29^, ^30^, ^31^ In addition, ILC2s may contribute towards increased type 2 cytokine production in the absence of the suppressive E‐cadherin ligation and decreased KLRG1‐E‐cadherin binding via the recently described mechanism of barrier sensing.^32^ Consistently, our results revealed that KLRG1 expression was significantly increased in the splenic lymphocytes of mice with DSS‐induced enteritis, whereas in vitro experiments confirmed that E‐cadherin could bind to KLRG1. Furthermore, significantly more Th2 type cytokines were secreted by ILC2s co‐cultured with mucosal epithelial cells with E‐cadherin silencing, suggesting that E‐cadherin expression in epithelial cells inhibits the function of ILC2s via a mechanism related to KLRG1. Therefore, we speculate that miR‐21a‐5p inhibits E‐cadherin expression, thereby reducing its binding to KLRG1 and indirectly promoting ILC2 activation to produce the excessive pathogenic Th2 type immune response typically observed in UC (Figure 5).

GATA‐3 is a key transcription factor that regulates the differentiation, maturation and activation of ILC2s and thus controls their number and function.^33^ In addition, studies have shown that sex hormones can affect KLRG1 and regulate the number and activity of ILC2s in lung tissue via GATA‐3.^34^ Our results confirmed that E‐cadherin supplementation in vitro significantly reduced the levels of Th2 type cytokines (IL‐5, IL‐13) secreted by ILC2s as well as their GATA‐3 expression. Previous studies have found that this pro‐activation can be blocked by silencing GATA‐3 in ILC2s,^31^, ^32^ suggesting that GATA‐3 plays an important role in ILC2 activation. Therefore, we speculate that reduced E‐cadherin expression in intestinal mucosa epithelial cells reduces its binding to KLRG1 on the surface of ILC2s, allowing KLRG1 to increase GATA‐3 expression in ILC2s and promote their activation (Figure 5). In conclusion, our results suggest molecular targets and provide a theoretical basis for elucidating the pathogenesis of UC and improving its treatment.

## CONFLICTS OF INTEREST

All authors have completed the ICMJE uniform disclosure form. The authors have no conflicts of interest to declare.

## AUTHOR CONTRIBUTIONS

**Jiaxi Lu:** Investigation (equal); Software (equal); Writing‐original draft (equal); Writing‐review & editing (equal). **Deliang Liu:** Conceptualization (equal); Project administration (equal); Resources (equal); Supervision (equal); Writing‐review & editing (equal). **Yuyong Tan:** Data curation (equal); Investigation (equal); Writing‐review & editing (equal). **Feihong Deng:** Investigation (equal); Methodology (equal); Software (equal). **Rong Li:** Conceptualization (equal); Data curation (equal); Formal analysis (equal); Funding acquisition (equal); Methodology (equal); Project administration (equal); Resources (equal); Supervision (equal); Writing‐original draft (equal); Writing‐review & editing (equal).

## Supporting information

Supporting information

## Data Availability

The data that support the findings of this study are available from the corresponding author upon reasonable request.
