# Supplementary material for: M1 Macrophage exosomes MiR‐21a‐5p aggravates inflammatory bowel disease through decreasing E‐cadherin and subsequent ILC2 activation
Source: J Cell Mol Med. 2021 Feb 10;25(6):3041–50. doi: 10.1111/jcmm.16348 (PMC7957199; doi:10.1111/jcmm.16348)
Supplement: Supplementary file 1 — Supporting information [file JCMM-25-3041-s001.docx]

Supplementary table 1. The primer sequences used in this study for RT-PCR

|  | Forward Primers(5’-…-3’) | Reverse Primers(3’-…5’) |
| --- | --- | --- |
| Human IL-4 | TCTCACCTCCCAACTGCTTC | TGTCTGTTACGGTCAACTCG |
| Human IL-5 | ATCTTTCAGGGAATAGGCACA | TTGCAGGTAGTCTAGGAATTGGT |
| Human IL-13 | CACTTGCCTTGGCGGCTTTG | CCTTCTGGTTCTGGGTGATG |
| Human IFN-γ | ACTTCTTTGGCTTAATTCTC | AGTTCCATTATCCGCTACAT |
| Human GATA-3 | GGAGTGTGTGAACTGTGGGG | TTCGCTTGGGCTTAATGAGG |
| Human GAPDH | GAGCCACATCGCTCAGACAC | CATGTAGTTGAGGTCAATGAAG |
| Mouse IFN-γ | ATCTGGAGGAACTGGCAAAA | TTCAAGACTTCAAAGAGTCTGAGGTA |
| Mouse IL-4 | TCGGCATTTTGAACGAGGTC | GAAAAGCCCGAAAGAGTCTC |
| Mouse IL-5 | ATGGAGATTCCCATGAGCAC | GTCTCTCCTCGCCACACTTC |
| Mouse IL-13 | CAGCTCCCTGGTTCTCTCAC | CCACACTCCATACCATGCTG |
| Mouse GAPDH | GATGCAGGGATGATGTTCTG | GTGAAGGTCGGTAACGG |
| Mouse E-cadherin | TTGGTGTGGGTCAGGAAATC | GTGTCCCTCCAAATCCGATAC |
| Human E-cadherin | ACGCTCGGCCTGAAGTGA | ATTCGTTCAAGTAGTCATAGTCCTGG |
| Human KLRG1 | CTCACACCTCCTTGTGATAAC | TTGTTCCTCAGACCAATCCA |
| miR-21a-5p | ACACTCCAGCTGGGTAGCTTATCA  GACTGAT | CTCAACTGGTGTCGTGGAGTCGGC  AATTCAGTTGAGGTCAACAT |
| U6 | CTCGCTTCGGCAGCACA | AACGCTTCACGAATTTG |


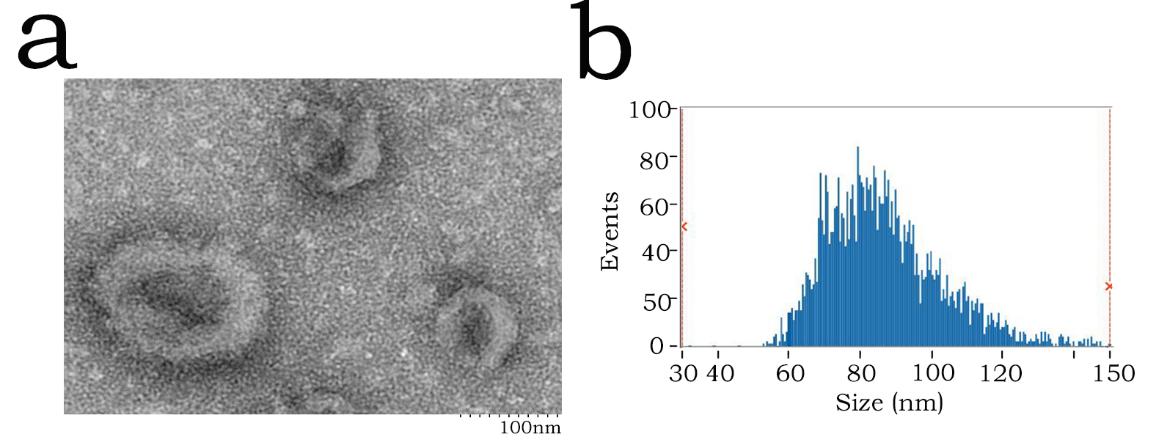


Supplementary figure 1 Identification of intraperipheal macrophage exosomes

（a）Exosomes were discoid vesica under transmission electron microscope with 30-80nm diameters; (b) Particle size analysis showed that the diameters of exosomes were mostly 60-120nm with a peak of 80nm.


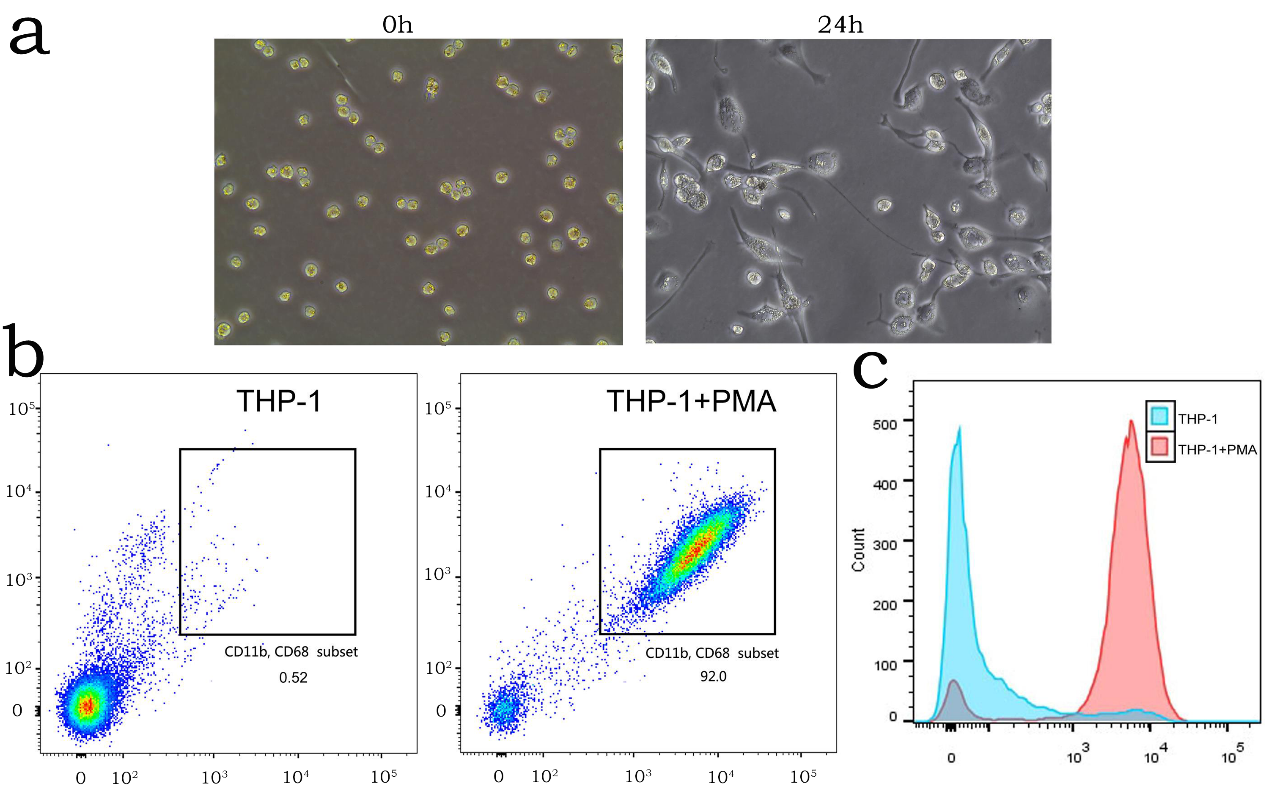


Supplementary figure 2 Identification of THP-1 induction

1. THP-1 changed morphology after treated with PMA (200×); (b) Successful rate of M0 induction was above 90% after treated with PMA; (c) CD11b expression increased after treated with PMA.


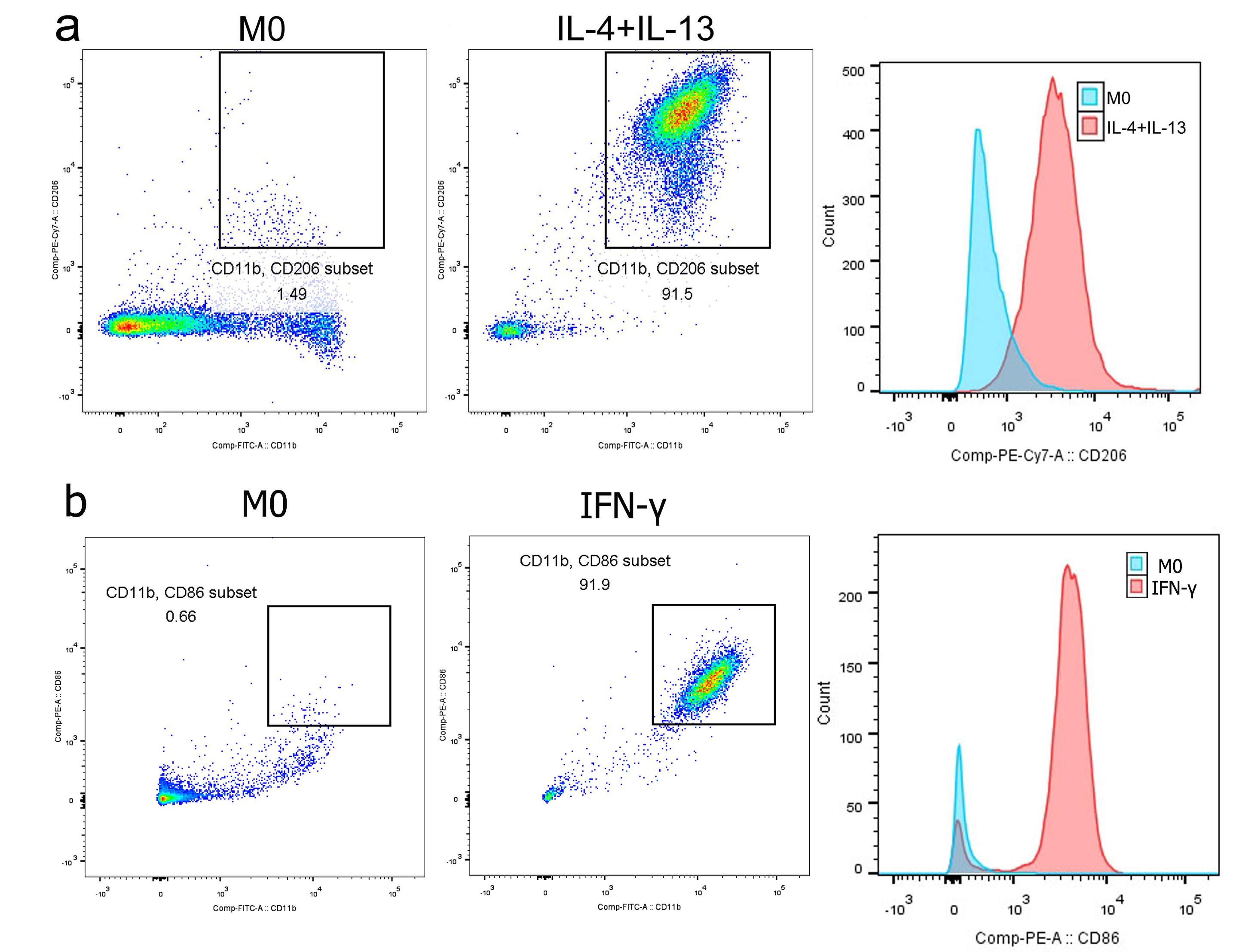


Supplementary figure 3 Identification of THP-1 induced M1 and M2 cells

1. Flow cytometry results of M0 cells before incubated with IL-4 and IL-13 for 18 hours (M0 group) and M0 cells after 18 hours incubation (IL-4 +IL-13 group). CD11b are used as a marker of macrophages and CD206 are used as a marker of M2 cells. The curve chart showed the CD206 expression of and M0 group and IL-4 +IL-13 group.
2. Flow cytometry results of M0 cells before incubated with IFN-γ for 18 hours (M0 group) and M0 cells after 18 hours incubation (IFN-γ group). CD11b are used as a marker of macrophages and CD86 are used as a marker of M2 cells. The curve chart showed the CD86 expression of and M0 group and IFN-γ group.


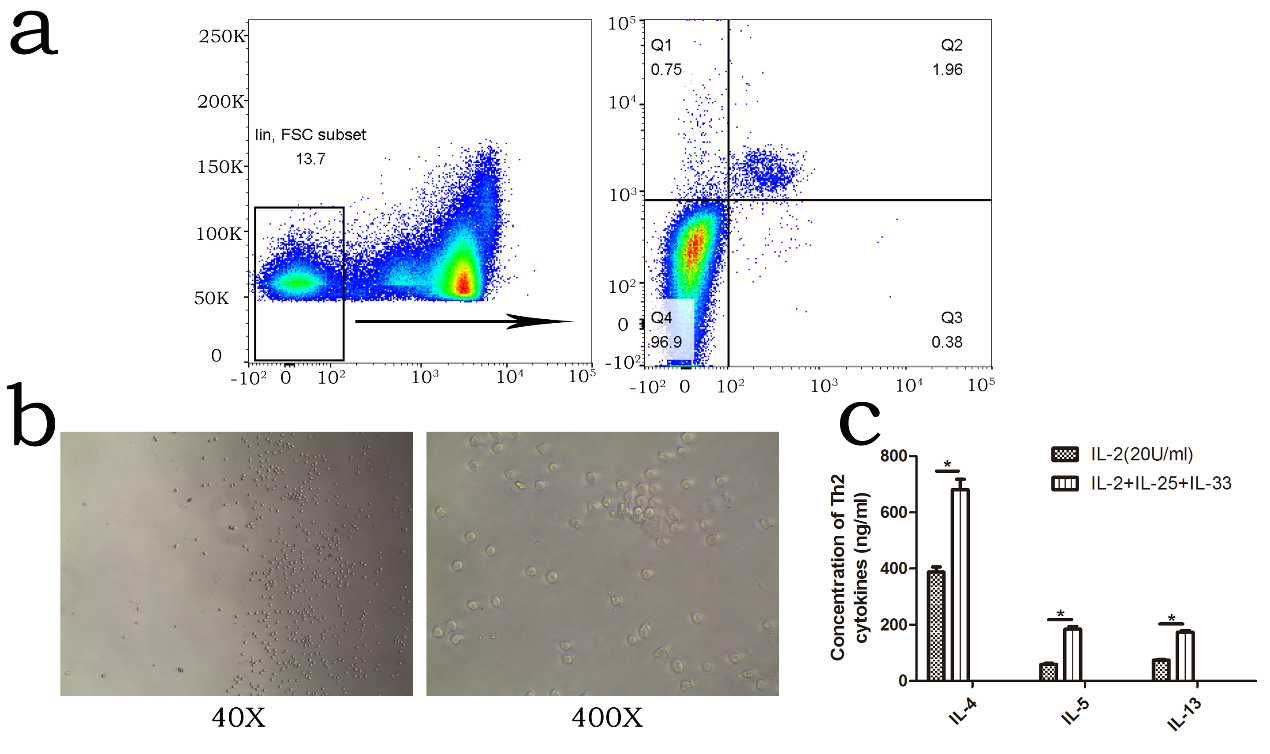


Supplementary figure 4 Characteristic under microscope and identification of flow cell sorting and ILC2 culture in vitro

1. PBMC were flow cell sorted to harvest ILC2; (b) Characteristic of ILC2 under white light microscope; (c) IL-25(0.4ng/mL)+IL-33 with different concentration treatment activated ILC2 in vitro. *P<0.05.


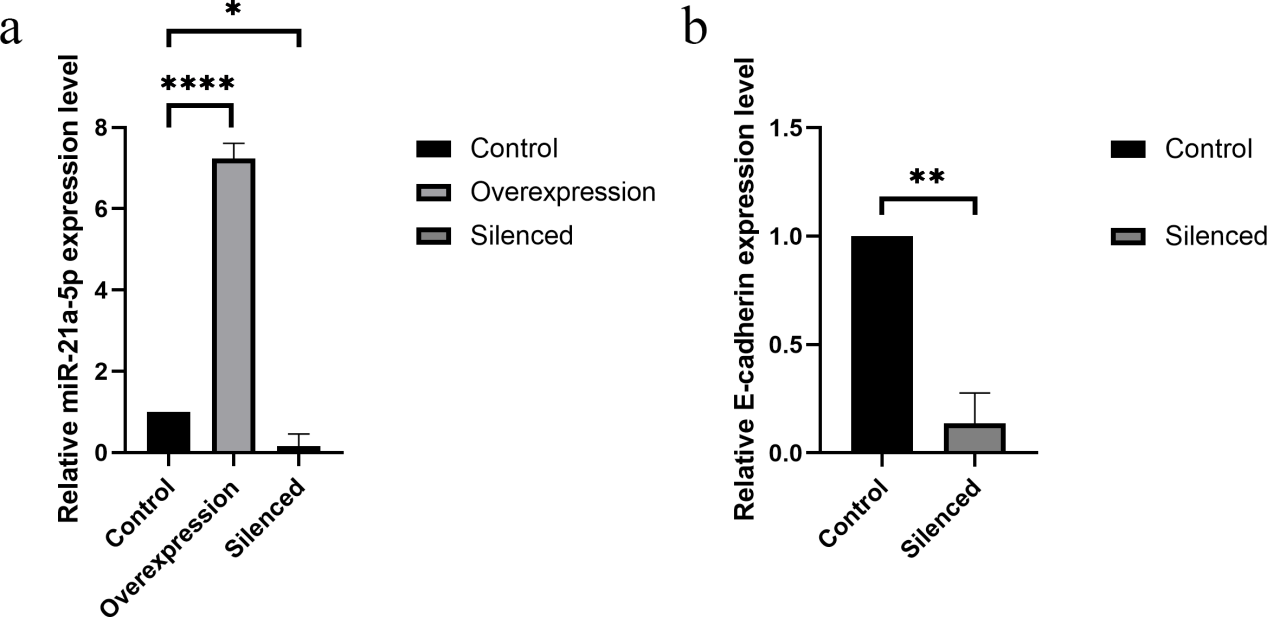


Supplementary figure 5 Confirmation of overexpressing and silenced model using RT-PCR.

(a)Relative expression level of miR-21a-5p in wild type, miR-21a-5p-overexpressing and -silenced FHC cell strains. (b) Relative expression level of E-cadherin in wild type and E-cadherin-silenced FHC cell strains.


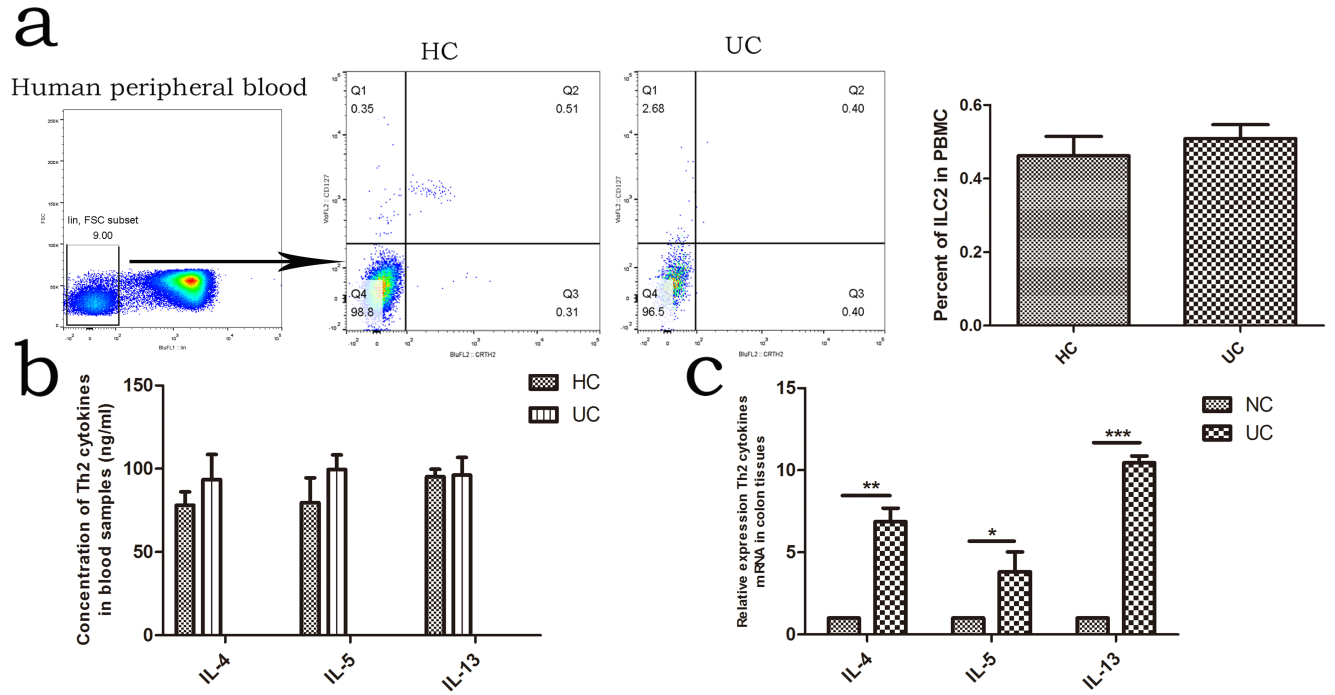


Supplementary figure 6

(a)The proportion of ILC2s in the peripheral blood of healthy volunteers and patients with active UC, (b) as well as the expression of Th2 cytokines. (c)Raletive expression levels of Th2 cytokine mRNA between two groups.
